# Rethinking the Current “Stage-and-Wait” Paradigm

**DOI:** 10.1017/S1049023X23000079

**Published:** 2023-04

**Authors:** Morel Ragoler, Irina Radomislensky, Eran Dolev, Liran Renert, Kobi Peleg

**Affiliations:** 1. National Center for Trauma and Emergency Medicine Research, Gertner Institute for Epidemiology and Health Policy Research, Sheba Medical Center, Tel Hashomer, Israel; 2. Tel-Hai Academic College, Upper Galilee, Israel; 3. Institute for Risk and Disaster Reduction, University College London, London, UK; 4. Department of Emergency and Crisis Management, The Israel Academic College in Ramat Gan, Ramat Gan, Israel

**Keywords:** explosions, mass-casualty incidents, safety, secondary explosion

## Abstract

**Introduction:**

The experience of terrorist incidents involving a secondary explosive device that targeted rescue forces led to changes in the safety protocols of these forces in most countries of the world. These protocols are the foundation of the current “Stage-and-Wait” paradigm that prohibits the entry of Emergency Medical Services (EMS) from entering the scene and treating casualties until it is deemed safe. These guidelines were established absent of an evidence-base detailing the risk to responders and the potential consequences to the injured on-scene. The lack of clarity is compounded by the fact that different situations, as well as operational considerations, such as the length of time until bomb squad arrival at the scene versus time of massive bleeding injuries, for example, impact outcomes must be taken into account.

**Objective:**

This study sought to shed light on this matter while employing an evidence-based approach exploring the investigations of the frequency of secondary explosion threats in terrorist attacks over the last 20 years and discussing some of the ethical challenges and ramifications ensuing. While this study does not propose an outright change to current guidelines, in light of the evidence gathered, an open review and discussion based on the findings may be beneficial.

**Methods:**

The Global Terrorism Database (GTD) was used as the data source of bombing incidents world-wide.

**Results:**

The results revealed that approximately 70 per-1,000 bombing incidents involved secondary explosions across regions and countries within the study period.

**Conclusion:**

This study emphasizes the need to rethink the current “Stage-and-Wait” paradigm by recommending brainstorming conferences comprised of multi-sectoral experts aimed at deliberating the matter. World-wide experts in emergency medicine, bioethics, and disaster management should cautiously consider all aspects of bomb-related incidents. These brainstorming deliberations should consider the calculated risk of secondary explosions that account for approximately 70 per-1,000 bombing incidents. This study highlights the need to re-examine the current versus new paradigm to achieve a better balance between the need to ensure EMS safety while also providing the necessary and immediate care to improve casualty survival. This ethical dilemma of postponing urgent care needs to be confronted.

## Introduction

### Background

In recent years, a debate emerged concerning the adequate approach to provide immediate life-saving treatment to casualties in mass-casualty incidents (MCIs) involving explosions in the terrorism context.^1^ The premise of this debate revolves around whether or not Emergency Medical Services (EMS) should provide immediate life-saving procedures to casualties or wait for the bomb disposal unit to declare the scene safe of any additional explosive devices before entering the scene.^1,2^ The latter is the common “Stage-and-Wait” paradigm^2^ adopted by many countries that EMS should delay their life-saving actions due to possible secondary explosions.^3–6^ This paradigm was applied in various bombing incident guidelines for many years.^7,8^

The primary justification for this current paradigm was that terrorists might use secondary attacks, including secondary explosions, to increase casualties and instill more fear. In such circumstances, the possibility of secondary attacks targeting first responders is a valid concern.^9^ Other studies also point to the threat posed to first responders from secondary devices or suicide bombers that detonated their explosive devices principally after the arrival of emergency services,^4,10^ as observed in prior experiences.^4^ For example, the suicide bombing attack at Beit Lid junction in Israel on January 22, 1995 involved a secondary explosion. This attack occurred when two suicide bombers detonated at a bus station, where the second bomber detonated his device upon the arrival of the first responders with the principal intent of targeting them.^11^ The Boston Marathon bombings in Boston, Massachusetts USA on April 15, 2013 is another example of a bombing incident involving numerous explosions. This incident involved two improvised explosive devices (IEDs) that exploded near the marathon’s finish line a few seconds apart.^12^ The duration time between the two explosions and the distance between them points to the possibility that this type of incident may not necessarily endanger first responders, such as EMS.^12^ Secondary explosion incidents also occurred in European countries. For example, the 2016 terror attack in Brussels (Belgium) included two explosions at the Brussels-Zaventem airport,^13^ and the 2010 subway bombing attack in Moscow (Russia) was initiated by two suicide bombers, as published by the *Guardian*.^14^

First responders and EMS provide important life-saving procedures that can make the difference between life and death to many casualties. It is widely agreed among clinicians and EMS providers that the rapid administration of these life-saving procedures is paramount to saving as many lives as possible in MCIs.^1,2,9,15–18^ On the other hand, EMS safety on the scene is of great importance to ensure response capacities. Therefore, decision making during MCIs can often lead to choices between conflicting values that may jeopardize the EMS’ capabilities to provide life-saving actions. These conflicts pose a great predicament in an already very stressful situation,^19^ especially when examining bombing incidents that involved only a single device.^20–22^ Single explosion incidents occurred in many places, including North America,^22^ the Middle East,^23^ and Europe, as observed, for example, in Lyon, France, bombing in 2019 involved a single bomb.^24^ These types of incidents may not pose similar threats to first responders as in the cases of secondary explosion incidents.

Rapid and immediate casualty care is crucial to saving lives and minimizing adverse outcomes, long-term complications of injuries for casualties,^1,2,9,15–18,25^ and reducing mortality.^2,9,15–17^ For example, severely injured victims (eg, suffering from hemorrhagic shock or blocked airways) may survive in greater numbers if provided with rapid, on-site life-saving procedures such as tracheal intubation and are immediately brought to definitive care.^25^

Dominique-Jean Larrey, the great French military surgeon of the Napoleonic Wars, introduced time into medicine at the beginning of the 19^th^ Century. It occurred when he realized that the period of time between injury and medical treatment was a crucial factor among the various considerations predicting the outcome of the injury in many casualties. In order to cut short this lag period, Larrey introduced into the medical service of the French army at the battlefield a special mobile medical unit. This unit, called “Ambulance Volante” – the Flying Ambulance, treated battle casualties close to the site of injury, at the proximity of the front line, sometimes under enemy’s fire, as soon as possible. Minimizing the lag period between injury and medical treatment improved significantly the outcome of many injuries.

The importance of time was gradually recognized beyond military medicine: it became a crucial issue also in civilian – general medicine, especially in surgery and in trauma.

When around a century later, the mechanism of hypovolemic shock became understood, it established the theoretical basis for the meaning of time for trauma victims and opened the gate to cope with hemorrhagic shock and to prevent its lethal consequences. Many years later, the leading trauma surgeon, R Adams Cowley, defined this lag period of time as “The Golden Hour:” the period of time essential for saving life. It was a part of his vision that: “the primary purpose of medicine is to save lives, that every critical ill or injured person has the right to the best medical care according to the state of art of medicine.”

Mass-casualty situations have challenged this professional attitude during the last decades. These previously unknown extreme events, caused mainly by terrorist activities, are characterized by many trauma victims simultaneously. It has been realized that in mass-casualty situations, the medical team was not able to give an appropriate treatment to every trauma victim. The cardinal limiting factor was time. Triage was the professional answer to the situation, backed by ethical principles of justice, beneficence, and nonmaleficence.

This adequate professional and ethical policy has been criticized since the second-half of the 20^th^ Century: in various cases, the terrorists managed to plant another bomb or a demolition charge at the site of the initial bomb, aiming at harming the rescue teams treating the victims at the bomb site. The necessity to protect the medical teams at a mass-casualty site has been the origin of the “Stage-and-Wait” policy.

The results of this research demonstrate quite clearly that the occurrence of such extreme events is quite rare. Applying the “Stage-and-Wait” policy in all mass-casualty situations means that terror victims might be approached by a medical team only after an unknown and unpredictable period of time; sometimes too late. This, clearly, might jeopardize immediate treatment needed to save the lives or limbs of many casualties.

The Code of Ethics for EMS Practitioners^26^ details what constitutes ethical conduct by practitioners in handling emergency care. While detailing several elements, it does not include any information or reference to events with potential harm to the care provider.

The complexity of EMS provider behavior in terrorist attacks stems from the uncertainty involved in the attack. The potential of a second explosion does not allow for a clear identification of the nature of the current risk of a terrorist attack. This is akin to the differentiation between a shooting event or an active shooting event, in which the threat is still on-going, according to the US Department of Homeland Security (Washington, DC USA).^27^

The dilemma of health professionals faced with physical harm and the well-being of their patients is not unique to EMS providers;^28,29^ however, the critical nature of injuries and the unique nature of care requires significant attention be placed when dealing with emergency care in uncertainty.

### Importance

The tension between the need to administer immediate medical care to casualties and the possibility of secondary explosions persists. Adini, et al reported that in a multi-national expert panel involving EMS experts from all five continents, one of the items that remained unconsented was EMS teams avoiding entry to a risk area until declared safe.^26^ A more careful look into the appropriateness of the current “Stage-and-Wait” paradigm is called for consideration.^2,17,18^ Perhaps a new paradigm, one in which EMS medical providers should not wait for the scene to be declared clear before administrating their life-saving actions is required.^2,17,18^

### Goals of This Investigation

This study’s objective is to evaluate the relevance of the current “Stage-and-Wait” paradigm by exploring the frequency of secondary explosion incidents risking first responders and characterizing their main features.

## Methods

### Study Design and Setting

This is a retrospective cohort study examining incidents of terrorist attacks involving explosions. Terror attacks involving explosions from the years 1970-2019 were collected from the Global Terrorism Database (GTD).^30^ This comprehensive database is published via the National Consortium for the Study of Terrorism and Responses to Terrorism (START; College Park, Maryland USA). The data collected for the GTD originate from diverse media sources categorized following the GTD protocol.^30^

### Data Collection and Processing

Overall, the GTD database has more than 200,000 global terror-related incidents reported. The data collection process included the following exclusion criteria: incidents reported prior to the year 2000 were excluded due to a large number (approximately 35%) of missing free-text summaries.

**Figure 1.**
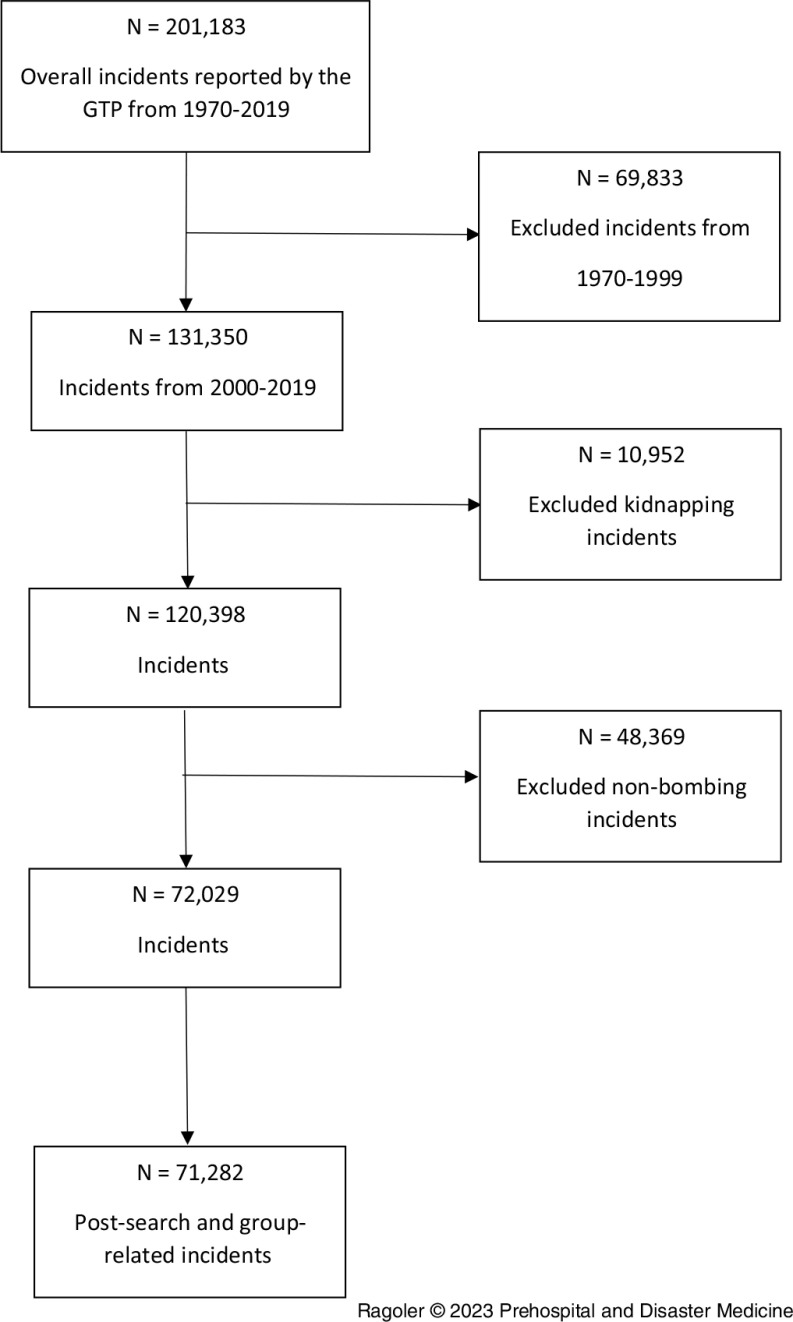
Description of the Incident Filtering Process Performed for this Study to Acquire Final Number of Incidents (n = 71,282).

The selection process of the relevant cases out of these 200,000 incidents reported in the GTD included several steps. This study focused on explosive-related incidents that occurred within the last 20 years. Hence, following the exclusion criteria above, all cases involving any type of explosives, including Molotov cocktail and Petrol bombs, were selected. The latter was classified as incendiary materials by the GTD database. However, the usage of these materials can lead to an explosion; thus, this is included (Figure 1).

Selected cases were screened for a selection of keywords. Keywords used to select cases with a secondary explosion included: *second*, *vehicles, bombs, rush, several, interval, investigate, crowd, respond, bombers, devices, gather, follow, another,* and *addition*. This stage aimed to identify typical phrases that may describe secondary explosions in a broad spectrum in the incident’s summary. Box 1 describes the phrases and classification methods conducted in this stage. The purpose of this step was to identify secondary explosions among the bombing incidents reported by the GTD. Secondary explosion incidents were defined as such if more than one explosive device was reported as present in the same vicinity or when the secondary explosion was linked to the initial incident according to the incident’s free-text summary.

**Box 1.**
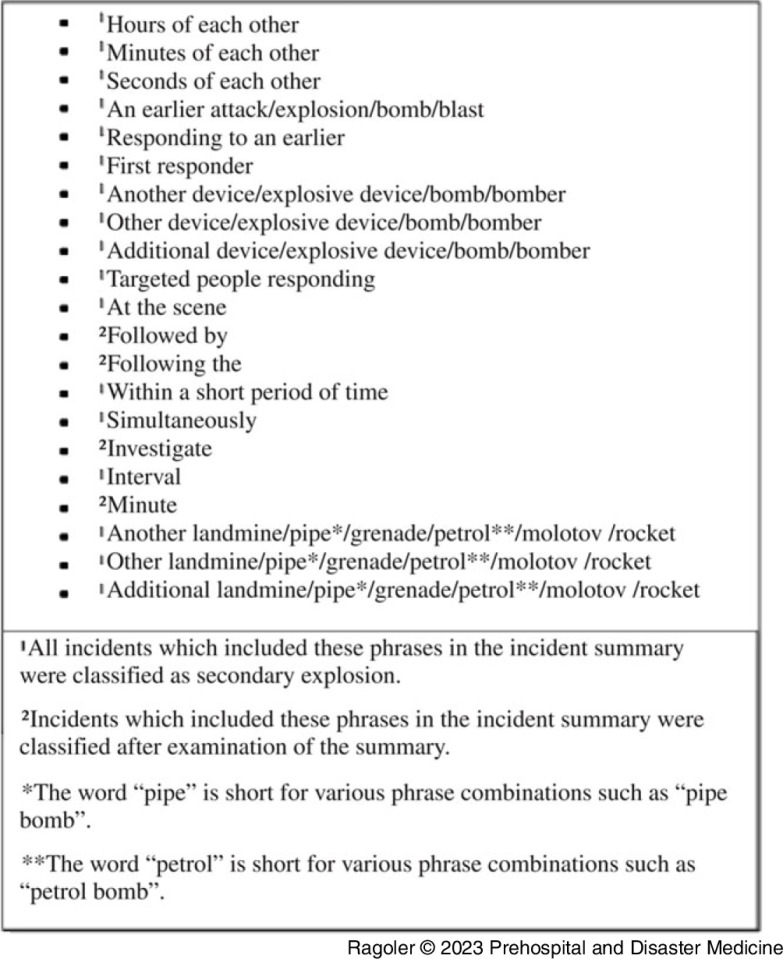
Phrases Used for the Third Search Stage in the Following Order.

Furthermore, related incidents listed as separate cases in which one of the cases reported as the secondary explosion of the other were grouped and classified as a secondary explosion incident. The single explosion incidents category included the following: (1) incidents involving a single explosive device, (2) incidents where several explosive devices detonated simultaneously, and (3) incidents in which the devices detonated in separate, remote, and unrelated locations rendering each explosion a separate incident, essentially. Related incidents listed as separate cases, in which none of the cases were reported as a secondary explosion, were classified as single occurrence. As described in the flow chart presented in Figure 1, the final sample size was 71,282 incidents.

### Variables

Several variables were collected for this study. First, filter variables that include the type of the event and the weapon used were collected. Second, investigated variables were collected, including the date, location (region, country, and city), a free-text summary of the incidents, indications for related incidents, successfulness classification of the incidents by the GTD (ie, a bombing incident was classified as “successful” when the bomb exploded), number of injured people, and death toll. The variable indicating the year of the incident was used to create the time periods assessed in this study. A casualty variable that sums the total number of casualties per incident (ie, number of injuries and deaths) was computed. The number of casualties was grouped based on the previously published MCI levels.^31^ This variable included the following groups: Level 1 = 1-10 casualties; Level 2 = 10-20 casualties; Level 3 = 20-100 casualties; Level 4 = 100-1,000 casualties; and Level 5 = over 1,000 casualties. Since the latter included only one incident in the final sample, Level 4 and Level 5 were grouped. Level 0 category was added to include incidents with no reported casualties.

### Analyses

All statistical analyses were performed using Microsoft Excel 2016 (Microsoft Corporation; Redmond, Washington USA) and RStudio version 1.4.1106 (Boston, Massachusetts USA).^31^ The statistical analyses included the following steps. First, the median of the secondary explosion incidents was calculated for the last two decades. Second, the number of secondary explosions per-1,000 was calculated for each decade and for the overall study period. Third, the number of secondary explosions per-1,000 was also calculated by regions and countries. Of note, the total number of explosion incidents in Australia was very low (15 incidents within a 20-year period), thus excluded. Furthermore, the countries-based analyses only included countries with a total of 10 and above explosion incidents. The fourth step included a comparison between the two types of bombing incidents regarding their success using the chi-square test.

Casualty-related analyses were also conducted for this study. The first analysis included comparing the MCI levels between the two types of bombing incidents using the chi-square test. The second casualties-related analysis included the ratio of deaths per number of incidents (ie, secondary and single explosions) observed from the years 2000-2019. Finally, the number of fatalities per secondary explosion successful incident was calculated for each decade. Of note, the causality-related analyses included measurements without missing values.

## Results

Approximately 57,200 (80%) of the final sample’s explosion incidents occurred from 2010 through 2019 (Table 1). Terror-related incidents originating from the Middle East & North Africa accounted for 31,511 (44.21%) of all investigated explosions, followed by Asia (27,751 [38.90%]), Africa (5,399 [7.57%]), Europe (4,748 [6.66%]), America (1,858 [2.60%]), and Australia (15 [0.02%]); Table 2.

**Table 1.** Number of Secondary and Single Explosion Incidents per 1,000 for each Decade

Type of Explosion	2000-2009	2010-2019	2000-2019
Secondary Explosion	70.18	67.93	68.37
Single Explosion	929.81	932.06	931.62
Total (N)	14,092	57,190	71,282

**Table 2.** Number of Secondary and Single Explosion Incidents per 1,000 Incidents Assessed by each Region^a^

Type of Explosion	Africa	America	Asia	Europe	Middle East & North Africa	Total
Secondary Explosion	80.2	71.58	69.07	60.02	66.67	68.37
Single Explosion	919.79	928.41	930.92	939.97	933.32	931.62
Total (N)	5,399	1,858	27,751	4,748	31,511	71,282

a A significantly smaller number of incidents (five secondary explosions of 15 bombing incidents) were observed in Australia, thus this region is excluded.

This study revealed that approximately 70 per-1,000 bombing incidents involved secondary explosions from the years 2000-2019. Similarly, 70.18 and 67.93 per-1,000 bombing incidents involved secondary explosions during the first (2000-2009) and the second (2010-2019) decades of the study period, respectively (Table 1). A relatively low ratio was also found between secondary explosions and the overall number of bombing incidents during one-half of the study period (Median = 0.0695).

**Figure 2.**
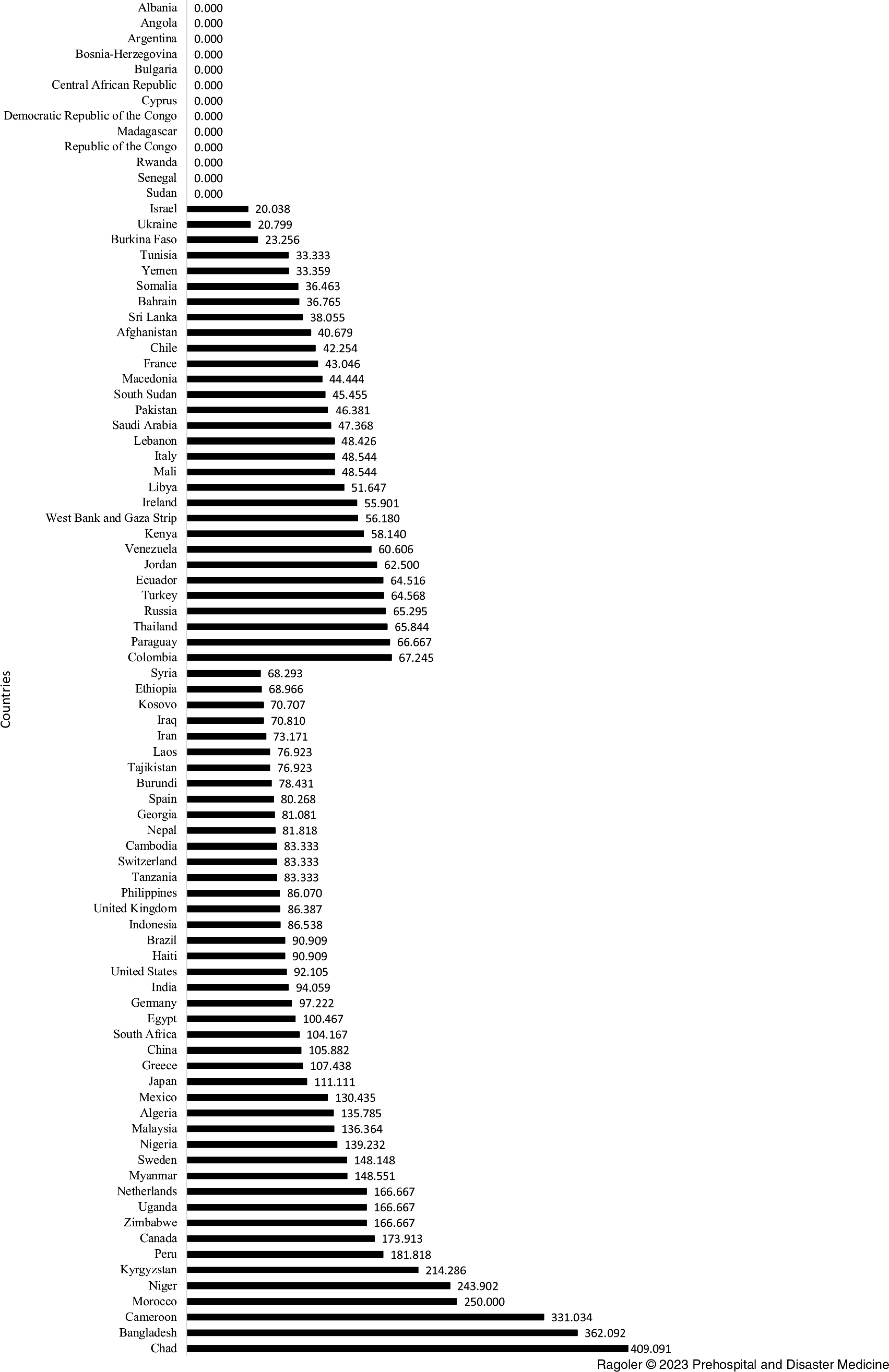
Number of Secondary Explosion Incidents per 1,000^a^ by Country from 2000-2019. ^a^ Part 2 includes countries in which 10(+) bombing incidents occurred during the study period.

The secondary explosion incidents were also analyzed across the five regions. As shown in Table 2, frequencies of incidents involving secondary devices were similarly low across the regions. From the years 2000 through 2019, a similar number of secondary explosions per-1,000 bombing incidents was found in Africa (80.20), America (71.58), Asia (69.07), Europe (60.02), and the Middle East & North Africa region (66.67). The total number of bombing incidents (15) reported in Australia was distinctly low, thus excluded (Table 2). Further investigations revealed the frequencies of the bombing incidents involving secondary explosions by country. These investigations included countries where ten and above bombing incidents occurred during the study period. As illustrated in Figure 2, the bombing incidents reported among 13 (approximately 15%) of the 88 investigated countries did not involve secondary explosions. More than one-half (75%) of the 88 investigated countries reported less than 100 per-1,000 bombing incidents involving secondary explosions from the years of 2000-2019 (Figure 2).

Most (3,593 [73.7%]) of the secondary explosion incidents were classified as successful (ie, the explosive device(s) detonated), according to the GTD. A slightly higher number (56,725 [85.4%]) of success was reported among the explosion incidents that involved a single explosive device (Table 3).

**Table 3.** The frequency of successful a and unsuccessful events among explosion incidents

Successful Incidents	Secondary Explosion Incidents	Non-Secondary Explosion Incidents	Total
	N (%)	N (%)	N (%)
Successful N (%)	3,593 (73.7%)	56,725 (85.4%)	60,318 (84.6%)
Unsuccessful N (%)	1,281 (26.3%)	9,683 (14.6%)	10,964 (15.4%)
Total	4,874	66,408	71,282

Note: GTD defines an explosion incident as “successful” if the explosive device detonated. P <.001.

Abbreviations: GTD, Global Terrorism Database.

As shown in Table 4, among the successful bombing incidents, Level 0 and Level 1 MCIs were more frequent among explosion incidents involving a single explosive device. However, Level 2 and above MCIs were more frequent in secondary explosion incidents. For example, Level 3 MCIs were more common (526 [14.6%]) among secondary explosion incidents than in explosion incidents involving a single explosive device (3,797 [6.7%]). Among incidents involving a secondary explosion, the deaths per number of successful incidents ratio was 4.93 compared with the 2.32 ratio found among the single explosion incidents. The number of fatalities per secondary explosion successful incident decreased across the investigated period (Table 5).

**Table 4.** Frequency of Casualties-Based MCI Levels among Successful Explosion Incidents

Casualties-Based MCI Levels	Secondary Explosion Incidents	Single Explosion Incidents	Total
	N = 3,593	N = 56,725	N = 60,318
Level 0	1,072 (29.8%)	15,124 (26.7%)	16,196 (26.9%)
Level 1	1,320 (36.7%)	29,444 (51.9%)	30,764 (51.0%)
Level 2	387 (10.8%)	5,254 (9.3%)	5,641 (9.4%)
Level 3	526 (14.6%)	3,797 (6.7%)	4,323 (7.2%)
Level 4	105 (2.9%)	379 (0.7%)	484 (0.8%)
Missing	183 (5.1%)	2,727 (4.8%)	2,910 (4.8%)

Note: GTD defines an explosion incident as “successful” if the explosive device detonated.

Levels are defined as follows: Level 0 = no casualties reported; Level 1 = 1-10 casualties; Level 2 = 10-20 casualties; Level 3 = 20-100 casualties; Level 4 = >1000 casualties. P <.001.

Abbreviations: GTD, Global Terrorism Database; MCI, mass-casualty incident.

**Table 5.** Number of Fatalities per Secondary Explosion Incident for each Decade

Fatalities among Successful Secondary Explosion Incident	2000-2009	2010-2019
Fatalities	6.344	4.479
Secondary Explosion Incidents	876	2,717

## Discussion

This study aimed to investigate the existing “Stage-and-Wait” paradigm that guides EMS personnel to delay life-saving procedures until the scene is declared safe from additional explosive threats in light of the probability of such threats. This study shows that after implementing very liberal inclusion criteria, the median ratio between secondary explosions and the overall number of bombing incidents was 0.0695 or less during one-half of the last two decades. Additional support for this notion is derived from the relatively low number of bombing incidents involving secondary explosions found during the study period (approximately 70 per-1,000 bombing incidents), which was similar across the investigated regions and countries. These broad criteria may include incidents where the explosions occurred simultaneously and may not endanger EMS personnel. Hence, this calculated risk of secondary explosions should be considered during the ethical brainstorming regarding the bomb-related paradigms.

Compared with other forms of trauma, terror victims sustain more severe and critical injuries.^32^ Multiple body regions injured in a single patient occurred in 62% of terror-related explosion victims.^33^ Experience from suicide bombings shows that nearly 30% of those harmed suffered severe to critical injuries (Injury Severity Score [ISS] ≥ 16).^34^ Additionally, a high prevalence of vascular trauma was evident in terror-related trauma as compared with non-terror-related trauma in a civilian setting. Patients with vascular trauma tended to be more complex. In the group of critically injured patients (ISS = 25-75), 51.4% had vascular trauma. Mortality rate among those suffering from vascular trauma was 22.9%.^35^ The profile of injury in mass-casualty events involving explosions thus emphasizes the importance of rapid medical attention.

Without a doubt, the dilemma being faced in this debate is complex and raises many theoretical and ethical questions. Most probably, there is no unequivocal answer. The discussion can be summarized into the following conceptual and policy questions: Is there a way to allow EMS to provide life-saving procedures alongside the vetting of the scene for secondary devices by the bomb disposal units that is both safe and effective? If it’s not possible, should a maximum waiting time for EMS be set, prior to entry, even if the scene has not yet been declared safe? If the bomb disposal unit is delayed to the scene for any reason, should EMS wait indefinitely before providing life-saving procedures to the injured?

The Code of Ethics for medical providers pledges personnel to “conserve life, alleviate suffering, promote health, do no harm, and encourage the quality and equal availability of emergency medical care.”^26^ The nature of injuries is such that following some time, sustaining life may no longer be possible. Placing responders in jeopardy without merit is not to be contemplated. The dilemma becomes evident when the injured on-scene are hemorrhaging in front of responders. The quantifiable risk to the responders is known, as is the risk to the injured. In these conditions, can the decision to provide medical care only after receiving an all-clear be absolute? If so, may that not open the prospect of a slippery slope to responders’ questioning whether they place their personal safety in providing immediate care to patients with limited prospects of survival? A step further may lead to their questioning patients’ quality of life after sustaining certain levels of injury.

Some articles indicate that first responders should initiate their response upon arrival on the scene,^2,17,18,36,37^ and even prior to the declaration of the scene as cleared by the bomb disposal unit.^26,38^ Nevertheless, the current “Stage-and-Wait” paradigm calling for medical care to be postponed until the scene is declared safe is common practice in many Western protocols for MCI management,^2,4^ especially in the United States^3^ and Europe.^6^ This current paradigm has existed for many years and is applied throughout various guidelines, such as those published by the US Department of Justice Office of Justice Programs (Washington, DC USA) in 2000^7^ and the US Centers for Disease Control and Prevention (Atlanta, Georgia USA).^8^ Indeed, this concern is echoed by the results of this current study in which most (approximately 75%) secondary explosion incidents reported by the GTD actually involved successful explosions and led to deadly results, more so than single explosion incidents. For example, approximately 30% of secondary explosion incidents were classified as Level 2 MCIs and above.

In a study performed by Adini, et al, a multi-national expert panel was engaged to explore consensus over different policies for managing EMS in MCIs. Of the 21 proposed policies, five were not consented by the minimum threshold required (70%). Among those unconsented policies was the policy to allow EMS to enter risk zones before being declared safe by the police. According to the authors, experts’ opinions differed according to the continent of origin. Experts from Europe, Asia, and Oceania were reluctant to endorse this policy, whereas experts from the Middle East, North America, and Africa were in favor of endorsing it. The findings of Adini, et al suggest that while some experts are hesitant toward a paradigm change in this regard, other experts were inclined to endorse such change.^37^

The results of this study support the notion to rethink the current “Stage-and-Wait” paradigm of delaying EMS from entering a scene of an explosion-based MCI until declared safe. As illustrated by the results, the number of fatalities per secondary explosion decreased over the last two decades. Hence, the ethical debate should also consider the notion suggested by several current guidelines that emphasize the revision of the “Stage-and-Wait” paradigm,^1,2,17^ even if such policies are perceived as risky for EMS.^1^ For example, the guidelines published by the US Department of Homeland Security in 2015^17^ highlighted the approach calling for immediate casualty care to prevent mortality.^17^ Some scholars also seem to agree with the need to change the current paradigm. For example, Smith, et al indicated that the current paradigm should be revised to allow emergency medical providers access to injured people within minutes of injury.^2^ Ashkenazi, et al also argue that casualties should be treated within the first 20 minutes.^36^ Other calls for paradigm change were voiced, for example, in a 2014 thesis published by Johnson and Thomas.^18^ Lastly, arguably, EMS personnel are usually operating with a culture that highly sanctifies the value of valor, as reported in mass-casualty events.^39^ In other words, they are willing to take risks to save other people’s lives.^39^

This study proposes that policymakers, EMS leadership, and experts in disaster management and bioethics should deliberate ethical questions regarding the calculated risk of secondary explosions while rethinking the bomb-related paradigms. Since there is no specific time limit for the arrival of bomb squads, life-saving casualty care may be delayed and result in losing causalities’ lives. Hence, novel approaches and actions should be discussed and examined in the context of terror-related bombing management protocols. These may include actions such as the EMS personnel having a free choice regarding entering the scene, creating well-trained EMS teams that are specialized in these types of incidents, and usage of arrival time estimation applications (eg, Waze [Google; Mountain View, California USA]) to assess the arrival time of the bomb squad for on-site decision making.

The directive that emergency medical teams will not enter the site of an explosion to treat casualties until the bomb squad has confirmed the area as safe is a decision that aims to save the lives of medical professionals and is fundamentally a right objective. However, in this formulation, it cannot be ignored that every minute for causalities with critical injuries without immediate life-saving treatment can result in death. In light of the data presented in this article, the relatively low risk of second explosions (approximately 70 cases in which there was a second explosion per 1,000 explosions) should be taken into account when deliberating all aspects of these paradigms.

An MCI, where upon arrival the medical teams are aware of potential injuries “bleeding to death” but are not allowed to approach and provide treatment because, according to the protocol, the medical teams must wait for the bomb squad to ensure that the area is safe, is a very sensitive and frustrating situation for first aid medical providers. In a massive bleeding situation, every minute is critical. Thus, as an example, in situations where the bomb squad’s estimated time of arrival is at least 15 minutes, for any reason, the authors would consider giving the medical manager at the scene the right to decide whether to approach, remove the injury to a safe area, and there treat with life-saving treatment. The medical staff could provide life-saving treatments during this time. Will the medical manager at the scene instruct the team to enter the dangerous area with him/her? The authors would predict that although most medical managers will not instruct the staff to follow into a zone not yet cleared of danger, many will choose to follow the leader when running in, and those who do not will not receive any negative feedback. Although the authors do not necessarily propose this code of behavior/protocol to be established, such possibilities should be considered.

## Limitations

This study has several limitations. First, the database used for the study is a convenience sample based on publicly available data and may not include all terrorist-related incidents. Second, the GTD database gathers data from various media sources, which may be incomplete despite the rigorous data collection protocols employed by the database. Third, this study included data from various countries that may differ based on several factors, such as type of government, demographic, and geographic characteristics, which may result in different outcomes of the bombing incidents.

## Conclusions

This article set out to examine the risks related to the issue of responder safety and the meaning of quality of care provided to patients, and to raise ethical questions regarding these problematic and complicated elements with the current unequivocal guideline governing delaying medical attention given a lack of certainty surrounding the possibility of an active threat. The findings of this study point to the need for brainstorming on this important issue with the participation of emergency medical professionals, bioethicists, public policy, and others on this critical and multifaceted issue.
